# Snaring and wildlife wastage in Africa: drivers, scale, impacts, and paths to sustainability

**DOI:** 10.1093/biosci/biaf014

**Published:** 2025-04-09

**Authors:** Sean Denny, Lauren Coad, Sorrel Jones, Daniel J Ingram

**Affiliations:** Bren School of Environmental Science and Management, University of California, Santa Barbara, Santa Barbara, California, United States; Center for International Forestry Research, Bogor, Indonesia; Department of Biology, University of Oxford, Oxford, England, United Kingdom; RSPB Centre of Conservation Science, Cambridge, England, United Kingdom; Durrell Institute of Conservation and Ecology, School of Natural Sciences, University of Kent, Canterbury, England, United Kingdom

**Keywords:** conservation, food waste, hunting, trapping, wild meat

## Abstract

Snaring is considered to be the most common form of hunting in Africa. Although snaring can provide hunters with valuable food and income, it can also devastate wildlife populations when practiced unsustainably and has significant animal welfare implications. Snaring can also be wasteful, both when animals escape with fatal injuries and when catch is discarded. In the present article, we argue that snaring is a regional-scale threat to wildlife and to the sustainable use of biodiversity in Africa. We show that snaring in Africa is geographically widespread and locally intense, that tens of millions of snares are likely set across the continent annually, and that, at a minimum, tens of millions of kilograms of wild meat are probably wasted in Africa every year because of snaring. We discuss opportunities to address these impacts through changes to governance and enforcement and by reducing urban demand for wild meat.

Humans have been hunting other animals for food for at least 2 million years (Bunn and Pickering 2010, Bunn and Gurtov 2014), but in the last few centuries, human population growth, advancements in hunting technologies, and the emergence and proliferation of commercial trading networks for wildlife have elevated hunting rates to unsustainable levels in many parts of the globe (Milner-Gulland et al. 2003, Lindsey et al. 2013, Benítez-López et al. 2019). This has caused widespread declines in wildlife populations, including regional and global extinctions (Dirzo et al. 2014, Benítez-López et al. 2017). Such declines threaten not only Earth's biodiversity but also human well-being (Wells et al. 2024). Millions of people across the planet hunt or consume wildlife for food or income (Golden et al. 2016, Ingram et al. 2021). Wildlife declines can also reduce ecosystem services and functions important to humanity, such as nutrient cycling, pollination, water provisioning, and the regulation of zoonotic diseases (Young et al. 2016). The importance of stemming wildlife loss for human well-being is reflected in international agreements and frameworks that call for the halt of biodiversity loss and the sustainable use of biodiversity, including the United Nations Sustainable Development Goals (United Nations 2015) and the Kunming–Montreal Global Biodiversity Framework (CBD 2022).

Globally, humans use a variety of tools and methods to hunt wildlife, but in Sub-Saharan Africa and in parts of Asia, snaring is thought to be the most common (Lindsey et al. 2013, Aziz et al. 2017, Gray et al. 2018, Coad et al. 2019, Gubbi et al. 2023, Haq et al. 2023). Snaring can be a cheap and effective way to obtain food and income (Lindsey et al. 2013), but it can also devastate wildlife populations when practiced unsustainably (Gray et al. 2018), leave animals injured when they escape from snares (Noss 1998), and be wasteful when hunters discard their catch because it has rotted in unchecked snares, been scavenged by another species, or the species caught is considered undesirable (Noss 1998, Kümpel 2006). As a regional-scale conservation issue, snaring has received significant attention in Asia (e.g., Gray et al. 2018, Belecky and Gray 2020, Tilker et al. 2024), but snaring also threatens the conservation and sustainable use of Africa's wildlife. Previous studies of snaring in Africa have been restricted to local contexts, specific ecosystem types or species, or only a subset of snaring's potential impacts. An overview of snaring in Africa that addresses its drivers and cumulative impacts can illuminate the scale of snaring in the region, highlight to policymakers and practitioners the need to manage snaring, and provide insights about how the negative impacts of snaring could be addressed.

In this article, we present an overview of snaring in Africa. We begin by explaining what a snare is and what drives snaring in Africa. We then discuss the scale of snaring in the region and its consequences for wildlife, both for wildlife populations and for individual animals. Next, we estimate the volume of *wild meat* (the meat from wild animals, excluding fish; Ingram et al. 2021)—and therefore wildlife—wasted in Africa every year because of snaring. We collate and present data on wastage in snaring from two dozen studies and review factors that influence wastage rates at local scales. We conclude by discussing ways to address the negative impacts of snaring, focusing on changes in governance and enforcement and on wild meat demand reduction in cities.

## What is a snare, and how are snares used in Africa?

A snare is a type of trap. Specifically, it is a noose that is placed in an area frequented by wildlife and tied to an *anchor*, often a tree, sapling, stick, or post placed firmly in the ground (Broom 2022). Sometimes the anchor is spring loaded, in which case it may be a flexible pole or sapling held out of its resting state with the help of an additional object (e.g., a stick; Dethier 1995). An animal becomes trapped in a snare when it places its foot or neck in the noose and then either pulls on the noose or displaces the spring-loaded anchor, both of which cinch the noose. Snares can be modified in size and strength to target species of different sizes, but they cannot distinguish between sex or age classes, nor between species of similar sizes (Noss 1995); as a result, snares can catch nontarget species (Becker et al. 2013), hereafter referred to as *bycatch*.

In Africa, snares are used to hunt mammals, reptiles, and birds (Noss 1995). They are typically placed along animal trails (Noss 1995), but they can also be placed at the edges of waterways (Holbech 1998), around watering holes or flowering trees (Becker et al. 2013), on branches and logs (Holbech 1998, Coad 2007, Golden 2009, Bene et al. 2013), and in and around agricultural fields (Dethier 1995). At larger scales, snaring in Africa, like hunting generally in the tropics (Benítez-López et al. 2017, 2019), is often correlated with proximity to human population densities, roads, and protected area edges (Hofer et al. 1996, Wato et al. 2006, Coad 2007, Lindsey et al. 2011, Watson et al. 2013, Moore et al. 2018, Nieman et al. 2019, Mudumba et al. 2021, Kendon et al. 2022).

Because of their ability to be modified in size, strength, and placement, as well as their largely indiscriminate nature, snares in Africa affect an extraordinary diversity of wildlife. Mammals that have been reported being caught in snares in Africa range in size from rodents and shrews that weigh less than half of a kilogram to elephants; they include ungulates, carnivores, and primates of all sizes—both ground-dwelling species and arboreal (tree-dwelling) ones—as well as pangolins, aardvarks, and hyraxes (Fitzgibbon et al. 1995, Noss 1998, Fa and García Yuste 2001, Golden 2009, Lindsey et al. 2015). Reptiles that have been reported being caught in snares in Africa include pythons, elapids (mambas and cobras), chameleons, crocodiles, tortoises, and monitor lizards (Noss 1998, Fa and García Yuste 2001); birds include ground-dwelling species (e.g., francolins and guineafowl), hornbills, parrots, eagles, vultures, and crows (Noss 1998, Fa and García Yuste 2001, Kaschula and Shackleton 2009, Dounias 2016). In short, because of the versatile use and nature of snares, nearly if not all of Africa's terrestrial and semiaquatic birds, mammals, and reptiles above half a kilogram are probably vulnerable to snaring (as either targeted catch or bycatch).

Snares in Africa were traditionally made with plant and animal materials (Dethier 1996, Fotso and Ngnegueu 1997, Yasuoka 2006, Dounias 2016), but beginning in the early to midtwentieth century, hunters began making snares with wire or nylon rope, following their introduction to Africa by Europeans (Noss 1995, Fotso and Ngnegueu 1997, Dounias 2016). This shifted the landscape of snaring in Africa because it made snares more effective (lethal), more wasteful, and probably much easier for individual hunters to maintain in large quantities (see below). Today, the vast majority of snares in Africa are made with wire (Lindsey et al. 2013, Coad et al. 2019).

## What drives snaring in Africa?

Snaring in Africa is driven by many factors but primarily the need for food and income. In many parts of Africa, fish and livestock can be unaffordable or unavailable because of limited market access and endemic diseases that make it difficult to raise livestock (Lindsey et al. 2013, Wilkie et al. 2016). In such cases, snaring can be an affordable way to obtain animal-source food (Fa and Brown 2009, Lindsey et al. 2013). There is also a large market for wild meat in parts of Sub-Saharan Africa, both because of a lack of animal protein alternatives and because wildlife is consumed for social and cultural reasons, including taste preferences and, in some urban environments, its luxury status (Ingram et al. 2021). In the absence of other livelihood options, especially paid employment, many people living in rural areas snare and sell wildlife to generate income (Noss 1995, Lindsey et al. 2013, Ingram et al. 2021). Most of these people are small-scale farmers or fishers who supplement their livelihoods with hunting, especially during seasonal lulls in labor requirements or times of unexpected financial hardship (Fa and García Yuste 2001, Schulte-Herbrüggen et al. 2013, Wright et al. 2016).

Snaring in Africa is also driven by human–wildlife conflict. Many small-scale farmers place at least a few snares in or around their fields to protect their crops (e.g., Dethier 1995, Vanwijnsberghe 1996, Kümpel 2006, Greengrass 2011, Alexander et al. 2015, Borgerson 2016). Similarly, livestock owners can set snares to protect livestock from predators (e.g., Borgerson 2016, White and Van Valkenburgh 2022).

As with other forms of hunting, snaring in Africa is also driven by social and cultural factors. For example, snares can be used in traditional foraging expeditions (Yasuoka 2006) and to obtain food used in traditional medicine and ceremonies (Subramanian 2012). Indeed, hunting in Africa (not just snaring) can be a way to bond socially, provide gifts, have fun, pass on or facilitate traditions, make political statements, and adhere to social norms (Mackenzie 1997, Yasuoka 2006, Dickman 2010, Dounias 2016). Given the widespread use of snares to hunt in Africa, it is very likely that snares are used to one degree or another to fulfill all of these functions.

Although economic, social, and cultural factors primarily drive snaring in Africa, two additional factors strongly contribute to it. One is the *de facto* open-access nature of wildlife in many parts of the continent. Historically, wildlife in Africa was managed through customary governance structures, but these were largely destroyed by colonial administrations, which often replaced them with legal frameworks originally designed in Europe to manage recreational hunting (Mackenzie 1997, Walters et al. 2015). A consequence of this is that much wildlife in Africa is currently owned by the state, and although many countries have detailed legal frameworks that regulate hunting, including snaring, they are not designed to support hunting for subsistence, economic, or traditional purposes (Coad et al. 2019). In efforts to remedy these issues, some African governments have created new laws, but they have sometimes layered them over old ones, creating sets of laws that can contradict each other and make the legality of hunting under certain circumstances ambiguous (Van Vliet et al. 2019). The result is that national frameworks for regulating hunting can be highly ineffective, and because wildlife is owned by the state, many communities lack a legal basis to exclude noncommunity members from overhunting wildlife. This disincentivizes wildlife management by communities themselves (Why spend time and energy managing a resource if someone else can overexploit it?), leading to largely unregulated, open-access scenarios that are taken advantage of by hunters of all kinds, not just those who use snares (Coad et al. 2019, Mavah et al. 2022).

The other factor that largely contributes to snaring in Africa is the practicality of modern snares as hunting tools, which makes them widely appealing to hunters. Wire and nylon rope are much stronger than plant and animal materials traditionally used to make snares, which makes animals caught in snares less likely to break them when trying to escape (Noss 1995, Dounias 2016). Moreover, wire can be easily reinforced by looping or twisting strands together to make snares several strands thick. These features allow hunters to retain more catch with modern snares (i.e., those made with wire or nylon) than they can with snares made of traditional materials. They also allow hunters to target large species through trapping without having to build more labor-intensive traps, such as pitfall or deadfall traps (Noss 1995, Dounias 2016).

Wire for making snares can also be procured easily, cheaply, and legally. To make snares, hunters often repurpose wire from relatively common objects, such as fencing, bicycle or car parts, or electrical material (Noss 1998, Lindsey et al. 2011, Becker et al. 2013, Mudumba et al. 2021). Even when hunters purchase wire, it is often very inexpensive, the costs can be recouped with the sale of a single animal (Noss 1998), and snares can be reused for several months or even years before they rust (Noss 1998, Fialla Foffou 2011, Teutloff et al. 2021). Wire to make snares is also much more affordable and accessible than guns. Like modern snares, guns are extremely effective hunting tools and used by many hunters in Africa (Lindsey et al. 2013, Ingram et al. 2025), but guns can be prohibitively expensive and require permitting and ammunition, which adds to costs. Several studies from West and Central Africa demonstrate the costs of wire snaring relative to gun hunting. At the times of these studies, a yard or meter of wire—enough to make several snares (Noss 1998)—cost around US$0.50, whereas rifles and shotguns cost anywhere from US$50 to US$600 (Noss 1998, Ondo Obiang 2001, Rieu 2004, Abugiche 2008, Allebone-Webb 2009, Jones 2020, World Bank 2024), a substantial portion of money in West and Central Africa where GDP per capita can be in the hundreds of US dollars (World Bank 2024). Gun cartridges cost around US$1 each (Noss 1998, Ondo Obiang 2001, Rieu 2004, Abugiche 2008, Allebone-Webb 2009, Jones 2020, World Bank 2024), the same price as materials to make several reusable snares.

Snares are practical for hunters for several other reasons. Snares are quieter than guns and operate remotely, which makes hunters less likely to be detected by law enforcement if they are hunting illegally. Their remote operation also allows hunters to conduct other activities simultaneously, such as farming or fishing, and unlike gun hunting, snares do not necessitate hunting at night, which can be relatively dangerous (Coad 2007, Kümpel et al. 2009).

## The scale of snaring in Africa

Snaring in Africa occurs on an enormous scale. It is both geographically widespread and, in areas with high hunting pressures, locally intense. It occurs widely across Africa's forests and savannas and in every major region of Sub-Saharan Africa (Fa and Brown 2009, Lindsey et al. 2013, Borgerson et al. 2019). At local scales, individual hunters can set dozens or even hundreds of snares at a time. Among commercial hunters, operating anywhere from 60 to 100 snares at one time appears to be common (e.g., Vanwijnsberghe 1996, Noss 1998, Kümpel 2006, Coad 2007, Teutloff et al. 2021), but some hunters can operate between 200 and 300 snares (Seino et al. 1998, Coad 2007, Fialla Foffou 2011, Vath 2014) or even up to 500 snares or more at a time (Jambiya et al. 2007, Jones et al. 2020). Studies from Central Africa have found that thousands of snares can be set within the hunting territories of individual villages at any one time (Kümpel 2006, Coad 2007). Similarly, thousands—even tens of thousands—of snares can be set within individual protected areas in Africa annually (table 1). No empirical estimates exist for numbers of snares set across all of Africa, but crude extrapolations suggest that well over 40 million snares could be set annually in Central Africa alone (box 1).

**Table 1.** The number of snares removed from 11 protected areas in eight countries in Africa between 1997 and 2023.

Protected area	Size (in square kilometers)	Snares removed (total)	Snares removed per year (average)	Data period	Source
Lomami National Park, Democratic Republic of the Congo	1936	2237	2237	2016	Fournier et al. 2023
Maasai Mara National Reserve, Kenya^a^	482	16,045	1689	2001–2010	Dublin and Ogutu 2015
Ruma National Park, Kenya	120	651	217	2006–2008	Kimanzi et al. 2015
Liwonde National Park, Malawi	548	54,818	6852	2015–2022	African Parks 2016–2023
Nkhotakota Wildlife Reserve, Malawi	1794	5457	780	2015–2021	African Parks 2016–2022
Odzala-Kokoua National Park, Republic of the Congo	13,500	151,249	12,604	2011–2022	African Parks 2012–2023
Akagera National Park, Rwanda	1120	6797	680	2010–2015	African Parks 2011–2016
Nyungwe National Park, Rwanda	1019	17,071	8536	2021–2022	African Parks 2022–2023
Kibale National Park, Uganda^b^	760	2290	654	1997–2000	Wrangham and Mugume 2000
South Luangwa National Park, Zambia^c^	13,775	2563	427	2005–2010	Becker et al. 2013
Savé Valley Conservancy, Zimbabwe	3450	84,396	10,550	2001–2009	Lindsey et al. 2011

*Note:* The protected areas were chosen based on their having publicly available data on snare removals, collectively representing multiple regions and ecosystem types within Africa, and collectively having similarly high numbers and densities of snare removals as protected area in Southeast Asia reported by Belecky and Gray (2020; see table 2 for a comparison). For each protected area, data were available for between 1 and 12 years; the average number of snares removed each year was calculated by dividing the total number of snares removed by the number of years in the data period and rounding to the nearest whole number. The data in the present table are presented in a format similar to that in Belecky and Gray (2020) for ease of comparison.

a Snare removals in Maasai Mara National Reserve occurred over 9.5 years, and data were only available for a portion of the reserve, whose size is listed.

b Snare removals in Kibale National Park occurred over 3.5 years.

c For South Luangwa National Park, snare removal numbers were only available at the level of both the park and the surrounding game management area (and therefore not at the level of just the park); therefore, the size reported includes both areas.

Box 1. **Estimating the number of snares set annually in Central Africa.**

By combining findings from previous research on hunting in Central Africa, it is possible to estimate a range for the number of snares set in the region annually. For example, several studies have estimated amounts of wildlife harvested (i.e., hunted) per year in Central Africa. These studies estimate that anywhere from 1.6 million to 4.6 million metric tons of wildlife is harvested per year (Ingram 2018). In addition, Ingram and colleagues (2025) recently collated data from 83 studies conducted across West and Central Africa to analyze regional hunting patterns. Drawing on their findings, we estimate that between 14% and 65% of animals hunted in Central Africa are caught with snares (see supplemental file S1 for further explanation). Several studies also report body masses separately for animals snared and for animals shot. Averaging values from these studies yields mean body masses of 3.95 ± 2.58 kg for animals snared and 5.32 ± 0.43 kg for animals shot, and a body mass ratio of 0.74 for animals snared to animals shot (3.95 kg ÷ 5.32 kg; see supplemental file S1 for further details). By combining these parameters, it is possible to estimate a coarse range for the number of snares set annually in Central Africa.

For instance, we can start by estimating the proportion of total biomass (i.e., the mass of wildlife) hunted annually in Central Africa that is caught using snares. Using the values above, we estimate a proportion of between 0.108 and 0.579 (i.e., 10.8% to 57.9%; see supplemental file S1). Next, we can combine these values with the most conservative estimate of total regional annual harvest (from Ingram 2018) of 1.6 million metric tons, to produce a range of 172.8 million to 926.4 million kg of wildlife harvested annually in Central Africa through snaring (0.108 × 1.6 million metric tons and 0.579 × 1.6 million metric tons, respectively). Finally, we can estimate how many animals, and therefore snares, would be required to obtain these harvest amounts from snaring by dividing them by the average body mass of snared animals calculated above, 3.95 kg. This produces a range of 43.8 million to 234.5 million snares set annually in Central Africa (172.8 million kg and 926.4 million kg ÷ 3.95 kg, respectively).

Though these numbers are already large, there are reasons to suggest that they could nonetheless be underestimates. For example, they do not include snares that are set by hunters but fail to catch animals. In addition, the values that we used for the proportion of snared animals (out of the total number hunted) do not incorporate hunting by forest hunter-gatherers (see supplemental file S1), but forest hunter-gatherers are more likely to use snares than other types of hunters (Ingram et al. 2025).

Although we use parameters that may have changed since they were first generated, we have no reason to suspect that absolute snare numbers in Central Africa have decreased over time. If anything, we think they could be increasing. Snare densities are often correlated with roads and human population densities (Yasuoka 2006, Coad 2007, Watson et al. 2013, Moore et al. 2018, Nieman et al. 2019, Mudumba et al. 2021), and both human populations and road networks are growing rapidly in Central Africa (Kleinschroth et al. 2019, United Nations 2022). Furthermore, some species are more resilient to hunting than others (because of high reproductive rates) such that they can persist in heavily hunted systems even when other species have become locally extinct (Peres 2000, Nasi et al. 2011). In Afrotropical forests, which dominate Central Africa, these species are often those targeted by snaring (Cowlishaw et al. 2005, Fa et al. 2005). Therefore, even where some species have been driven locally extinct by hunting, snaring probably persists. All of this suggests that at a minimum probably over 40 million snares are set in Central Africa annually.

In certain parts of Africa, snare numbers and densities are probably comparable to those in parts of Southeast Asia, where conservation scientists consider there to be a snaring crisis (Gray et al. 2018, Tilker et al. 2024). For example, Belecky and Gray (2020) compiled data on numbers of snares removed by ranger patrols from 11 protected areas in five countries in Southeast Asia between 2005 and 2019; data were available for between 3 and 10 years per protected area. In total, 371,056 snares were removed from the protected areas, or if dividing by the sum of the number of years for which data were available per protected area, just over 4800 snares per protected area per year (Belecky and Gray 2020). By searching the peer-reviewed literature and available nongovernmental organization reports, we were able to find snares removed in similar numbers and densities from 11 protected areas in eight African countries between 1997 and 2023 (tables 1 and 2). It is important to note that in protected areas with snare removal programs, patrol coverage can be patchy because of logistical and funding constraints, and even at relatively high rates of search effort, large proportions of snares at patrolled sites can go undetected (even over half; Ibbett et al. 2020). Therefore, as Gray and colleagues (2021) note, snare removal numbers are often the “tip of the iceberg” relative to total numbers of snares set, and the numbers in table 2 probably represent a significant underestimate of true snare densities. Nonetheless, we believe that the similar numbers and densities of snares removed from these two sets of protected areas illustrates that the magnitude of snaring in parts of Africa could be similar to that in parts of Southeast Asia.

**Table 2.** Summary statistics for two snare removal data sets, one for select protected areas in Africa (see table 1) and the other for select protected areas in Southeast Asia (Belecky and Gray 2020).

Region	Number of protected areas	Data period	Total protected area-years	Total number of snares removed	Average number of snares removed in each protected area per year	Total combined area of protected areas (in square kilometers)	Average number of snares removed per square kilometer per year
Africa	11	1997–2023	70	343,574	4908	38,504	1.402
Southeast Asia	11	2005–2019	77	371,056	4819	37,406	1.417

*Note:* The snare removal numbers and densities were broadly similar in the two data sets. The data period is the collective time period over which all data were originally collected. Within this time period, data were available for only a subset of years for each protected area. Protected area-years is the sum of those numbers of years. See supplemental file S2 for further explanation of the calculations.

## The impacts of snaring on African wildlife

Snaring is a major driver of wildlife population declines across Africa. Over the last half century or more, overhunting has dramatically reduced mammal and bird populations in many parts of Africa, and snaring, in combination with gun hunting, is thought to be largely responsible for these declines (Mackenzie 1997, Lindsey et al. 2013, Benítez-López et al. 2017, 2019, Coad et al. 2019). Although in some cases it can be difficult to isolate the role of snaring (because multiple hunting methods often occur simultaneously in any one landscape), many studies have robustly linked snaring to wildlife declines across the continent. For example, studies have implicated snaring in declines of large herbivores or carnivores in Mozambique (Bouley et al. 2021), Tanzania (Hofer et al. 1996, Dublin and Ogutu 2015, Strauss et al. 2015), Uganda (Montgomery et al. 2023), Zambia (Banda et al. 2023), and Zimbabwe (Lindsey et al. 2011, Loveridge et al. 2020). Fitzgibbon and colleagues (1995) linked snaring to declines of small mammals and primates in parts of Kenya. Other studies have combined estimates of wildlife densities with both harvest rates (i.e., the number of animals killed in a given time period and area) from snaring and species-specific reproductive rates to show that snaring was unsustainable for wildlife populations. For instance, using these methods, snaring was found to be either unsustainable or likely unsustainable for antelope populations in Central Africa (Lahm 1993, Fotso and Ngnegueu 1997, Noss 1998, Fa and García Yuste 2001, Yasuoka 2006) and for lemur populations in Madagascar (Golden 2009). Furthermore, in a meta-analysis of hunting patterns across Afrotropical forests, Fa and colleagues (2005) found that hunters transitioned to targeting smaller species after snaring had depleted larger ones, underscoring snaring as a common driver of wildlife declines (and also, by disproportionately affecting larger-bodied species, of changes in the composition of wildlife communities). Snaring is also increasingly thought to be one of the most important threats to Africa's large carnivores, both because it kills large carnivores directly and because it depletes their prey (Becker et al. 2013, 2024).

**Figure 1.**
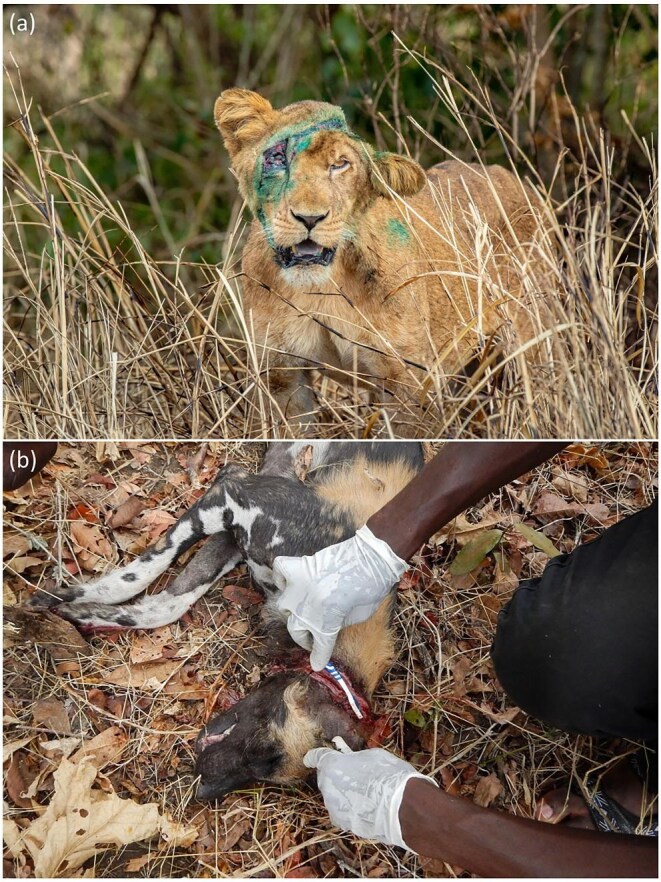
Animals with snare wounds. (a) A lion (***Panthera leo***) in Queen Elizabeth National Park, Uganda, after having a wire snare removed from its face and the wound treated. Photograph: Steve Winter. (b) An African wild dog (***Lycaon pictus***) in the South Luangwa Valley, Zambia, having a wire snare removed from its neck. The wire was deeply embedded in its tissue, creating an open wound. Photograph: Matthew Becker.

Beyond driving population declines, snaring in Africa can cause animals to experience particularly stressful deaths or, if they escape, to live with injuries. When caught in a snare, an animal can experience hours if not days of dehydration, starvation, hypothermia, or other extreme physiological stress from being exposed to nonhuman predators before being found by a hunter or otherwise succumbing to death (Broom 2022). When trying to escape from snares, animals can dislocate or break their bones, drive wire deep into their tissues, severing nerves and tendons, or amputate body parts (Noss 1998, Wrangham and Mugume 2000, Waller and Reynolds 2001). If an animal does escape from a snare, it does so by breaking the noose, breaking the snare free of its anchor, or through amputation. Many animals that escape from snares probably die shortly thereafter because of the severity of their wounds (e.g., because of blood loss, infection, or predation; Noss 1998, Kümpel 2006, Banda et al. 2023), but others survive. In Africa, great apes and other primates (Byrne and Stokes 2002, Beamish and O'Riain 2014), large carnivores (White and Van Valkenburgh 2022), and ungulates ranging from small forest antelopes to giraffes and elephants (Kümpel 2006, Obanda et al. 2008, Kasozi et al. 2023) have all been observed living with injuries from snares or otherwise having escaped from snares and survived. Many of these animals live with wire cinched around their bodies—their necks, legs, torsos, mouths, faces (figure 1), reproductive organs (Benhaiem et al. 2023), or trunks (Obanda et al. 2008). Some animals can be treated through veterinary intervention (figure 1; e.g., Haggblade et al. 2019, Banda et al. 2023), but such treatment is probably only available for a small subset of mainly large, charismatic mammals living in well-funded protected areas. Of the animals that escape from snares and survive, many live with permanent disabilities, such as missing or paralyzed hands or feet (e.g., Waller and Reynolds 2001, Beamish and O'Riain 2014).

Injuries from snares can be surprisingly prevalent among individuals in wildlife populations. In Uganda, for instance, nearly 20% of individuals (10 of 52) in a chimpanzee population had injuries from snares or other traps (Waller and Reynolds 2001). In an area over 1000 kilometers squared in the Central African Republic, over half of adult mammals, excluding elephants, were estimated to have injuries from snares (Noss 1998). At three sites in Zimbabwe, 26% to 44% of hyenas observed in camera traps had signs of snare injuries (Loveridge et al. 2020). For all of these reasons, snaring in Africa is also an animal welfare issue.

An important question from a conservation and ecological standpoint is whether permanent injuries from snares can affect individual fitness and, if so, scale up across individuals to affect population-level processes such as recruitment or evolution. Snaring could affect evolution if certain physical or behavioral traits resulted in some individuals in populations being disproportionately vulnerable to or affected by snaring (e.g., if some individuals had a greater propensity to migrate out of protected areas into locations where snare densities were higher). Although there is ample evidence that snare injuries can affect animal behavior (Kasozi et al. 2023), as far as we know, only one study has evaluated whether snare injuries can affect individual reproduction or long-term survival (and therefore fitness): In the Serengeti, female hyenas with snare wounds had longevity similar to that of hyenas without snares wounds but later ages of first reproduction, smaller litter sizes, and reduced offspring survival (Benhaiem et al. 2023). Other research, however, provides suggestive evidence that snare injuries can affect fitness. For instance, Mitani and colleagues (2024) noted that chimpanzees who were snared at a young age grow up to be smaller than chimpanzees who were not, which could affect their reproductive fitness. Yersin and colleagues (2017) found that chimpanzees with snare injuries had a higher prevalence of intestinal parasites than those without them, which also could affect reproductive fitness. Collectively, these studies suggest that injuries from snares could have important but as yet unappreciated impacts on African wildlife populations.

## Snaring and wildlife wastage

From both a conservation and a food systems perspective, one of the most concerning aspects of snaring in Africa is that it is wasteful. Wastage in snaring occurs both when hunters discard catch and when animals escape from snares with fatal injuries and die without being collected by hunters. Hunters discard catch either because it has rotted beyond salvage in snares left unchecked for too long, because it has been scavenged by another species, or because it is undesired bycatch (Noss 1998, Kümpel 2006, Coad 2007).

Although wastage is a known issue in snaring in Africa, its prevalence and magnitude have remained largely unexplored. To address this gap, we collated data on wastage rates in snaring from studies from across the continent. We then combined this data with previously published estimates of wildlife hunted for food (i.e., harvested) in Africa to estimate the amount of wild meat wasted annually in Africa because of snaring.

To collate data on wastage rates, we searched the literature for studies that had data on either discard rates or escape rates in snaring. We defined discard rates as the number of animals discarded out of the total killed by snares and escape rates as the number of animals that escaped out of the total caught, whether killed or escaped. We found 24 studies that measured discard rates, 3 of which also measured escape rates (see supplemental file S3 and supplemental table S2 for search methods and a list of the included studies, respectively). Five studies reported the proportion of catch found rotten without specifying the amount discarded. This distinction matters because not all catch that is found rotten in snares is discarded: Catch that has only just recently begun to rot can still be consumed, gifted, or sold (Kümpel 2006, Coad 2007). Therefore, studies that report the proportions of catch found rotten without specifying the amount discarded provide upper bounds of discard rates. We retained these studies because the proportion of catch found rotten is often highly correlated with discard rates (Coad 2007) and is therefore still useful in illuminating wastage in snaring.

**Figure 2.**
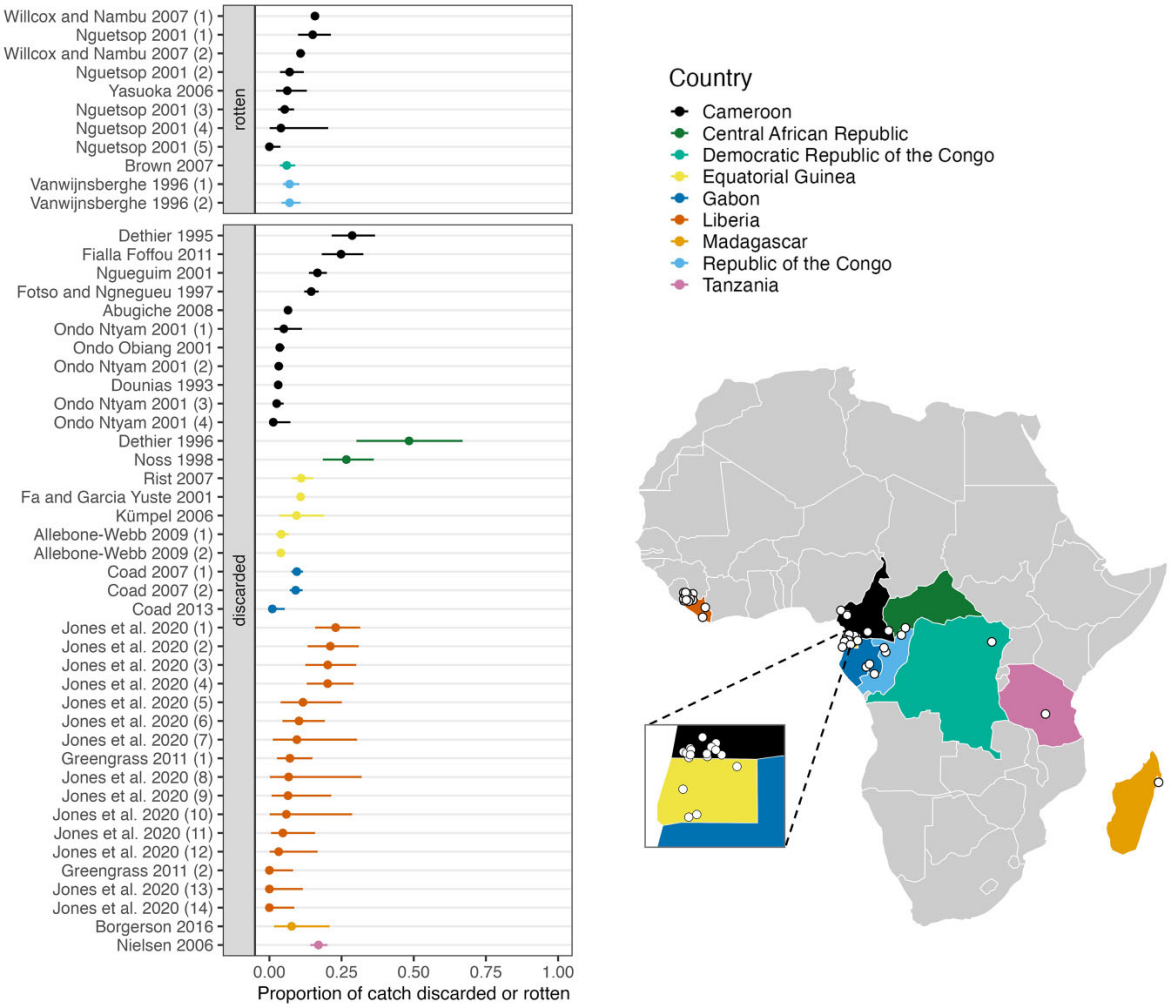
The rates at which catches from snares were reported discarded or rotten from 24 studies spanning 47 sites and nine countries in Africa. Each row in the plot is a different site, and the *y*-axis label is the study that included the site (see table S1 for a list of the studies). Some studies had data for multiple sites, in which case we indexed sites within the study and included a row for each site. Most sites (rows) correspond to a single village but, in some cases, to multiple villages (up to six). In each row, the point corresponds to the number of animals rotten or discarded out of the total number of animals killed by snares. The line overlapping the point is a 95% confidence interval. The plot is separated into the discarded and rotten categories because some studies only reported the proportion of catch found rotten without stating whether it was ultimately discarded. The map shows the countries and locations of the study sites. To protect the sites’ identities, their locations are slightly jittered.

Collectively, the 24 studies measured discard rates at 47 sites in nine countries and escape rates at 3 sites in three countries. The discard rates varied dramatically across sites and studies, from 0% to 48.4% of the catch (figure 2). The escape rates were more consistent, at around one-third to one-half of catch. Specifically, the escape rates were 36% (30 of 83 animals) at a site in Equatorial Guinea (Kümpel 2006), 38% (64 of 170 animals) at a site in the Central African Republic (Noss 1998), and 51% (101 of 198 animals) at a site Cameroon (Yasuoka 2006).

To estimate annual wastage across Africa because of snaring, we first used the information obtained in our literature review to estimate an average discard rate for all of Central Africa. We focused on Central Africa at this stage for two reasons. First, most of the available data on discard rates comes from Central Africa (figure 2). Second, regional estimates exist for amounts of wildlife harvested annually in Central Africa. Combining these parameters—an average discard rate for Central Africa and regional amounts of harvested wildlife—allowed us to estimate amounts of wild meat discarded annually in Central Africa because of snaring. Next, we carefully considered what this estimate could reveal about amounts of wild meat wasted annually across the entire continent, drawing on data on snaring and wastage from other parts of Africa.

For an average discard rate for Central Africa, we chose 9% (see box 2 for an explanation). Using this value as a starting point and cautiously extrapolating to the broader continent, we estimate that at a minimum tens of millions of kilograms of wild meat are wasted annually across Africa because of snaring (box 3). Whether wastage from snaring elevates hunting rates and, in turn, additively affects the sustainability of hunting systems in Africa is unknown. However, considering that snaring can drive wildlife population declines, reducing this level of unnecessary mortality undoubtedly would serve to alleviate pressures on wildlife, lead African hunting systems closer to sustainability, increase food security, and bolster opportunities for nature-based economic development.

Box 2. **What is an average discard rate for snaring in Central Africa?**

Discard rates in Central Africa are not systematically monitored and therefore it is not possible to answer this question definitively. However, using available data (figure 2), we estimate a mean discard rate of 9% for the following reasons:

First, calculating a weighted mean of discard rates from Central Africa—weighted by the number of animals killed in snares, to ensure that studies with relatively small sample sizes do not disproportionately influence the mean—yields a rate of 8.4% (1045 animals discarded out of 12,384 animals killed in snares; *n* = 15 studies encompassing 19 sites).

Second, calculating a weighted mean of all available discard rates (i.e., including those from outside Central Africa) produces a rate of 9.1% (1271 animals discarded out of 13,946 animals killed in snares; *n* = 19 studies encompassing 37 sites).

Third, several factors affect discard rates at local scales, which likely vary across hunters and over time. For example, many hunters who use snares also farm or fish, and during times of the year when these activities are more labor intensive, they can prioritize them over checking snares, leading to greater discard rates (Dethier 1995, Vanwijnsberghe 1996, Muchaal and Ngandjui 1999, Ondo Ntyam 2001, Brown 2007, Coad 2007). As a result, we suspect that studies that measured discard rates across a greater number of hunters and over longer time periods yielded discard rates that more accurately reflect what a yearly regional average discard rate might be for Central Africa. Studies from Central Africa that measured discard rates either over a year or across at least 30 hunters had a weighted mean of 8.5% (474 animals discarded out of 5604 animals caught in snares; *n* = 4 studies encompassing four sites).

Finally, in the studies in our data set, discard rates were measured in one of two ways: either by interviewing hunters after they returned from trips or by accompanying hunters on trips and recording discards through direct observation. In the former method, hunters can underreport discards if they consider it shameful to waste wildlife or fear retribution from conservation organizations (Jeanmart 1998, Coad 2007, Willcox and Nambu 2007). Because the majority of studies in our data set obtained data by interviewing hunters (*n* = 12 studies from Central Africa and *n* = 15 studies overall), we suspect that the means we report above (ranging from 8.4% to 9.1%) are underestimates of true discard rates. For all of the above reasons, 9% seems like a reasonable if not conservative average discard rate for Central Africa.

Box 3. **How much wild meat is wasted in Africa every year because of snaring?**

To address this question, we estimated at a coarse level the amount of wild meat discarded annually in Central Africa and then cautiously extrapolated our findings to the entire continent. We began, as we did in box 1 to estimate regional snare numbers, by drawing on previous research from Central Africa to estimate the amount of wildlife harvested annually in the region from snaring. Based on the most conservative estimate of total regional annual harvest, we estimated a range of 172.8 million to 926.4 million kg of wildlife harvested annually from snaring (box 1). It is not clear if the original estimate of total harvested wildlife that we used to generate this range included discarded catch. But if we conservatively assume that it did, we can apply our estimated average 9% discard rate for snaring in Central Africa (box 2) to the lower and upper values to generate a range of 15.6 million to 83.4 million kg of wildlife discarded in Central Africa every year because of snaring (172.8 million kg and 926.4 million kg × 0.09, respectively).

Next, because not all parts of animals are consumable, to convert this estimated volume of discarded wildlife into consumable wild meat, we applied a previously generated conversion factor of 0.7 (Fa et al. 2002) to our estimated range of discarded wildlife, above, to generate 10.9 million to 58.4 million kg of consumable wild meat discarded in Central Africa annually because of snaring (15.6 million kg and 83.4 million kg × 0.7, respectively). These numbers still do not account for animals that escape from snares with fatal injuries. But as we have shown in the main text, one-third to over one-half of animals can escape from snares, many with severe injuries that could be fatal, adding to wastage. This suggests that the amount of wild meat wasted annually in Central Africa because of snaring could exceed our upper estimate of 58.4 million kg.

Finally, to cautiously extrapolate these numbers to other parts of Africa, we considered rates of snaring and wastage in other parts of Africa. As we have shown, snares can be set in very high numbers outside of Central Africa (table 1). Discard rates can also be high outside of Central Africa (figure 2). Furthermore, it is possible that wastage rates could be much higher in parts of East or Southern

Africa where protected areas may be more regularly patrolled, which could result in hunters checking snares less regularly or abandoning them altogether if checking snares risked punishment. In Savé Valley Conservancy in Zimbabwe, for instance, nearly 60% of carcasses found in snares by ranger patrols were rotten or scavenged (Lindsey et al. 2011); in Murchison Falls National Park in Uganda, the number was also close to 60% (Mudumba et al. 2021). There is no scientifically rigorous comparison of wildlife harvest or snaring rates across regions of Africa, but even if one-half of total wildlife harvest from snaring in Africa comes from Central Africa, and average discard rates outside of Central Africa were also 9%, based on our calculations this would result in 21.8 million to 116.8 million kg of consumable wild meat discarded every year across Africa because of snaring (10.9 million kg and 58.4 million kg × 2, respectively).

Given that we used conservative parameters to calculate this range, including the lowest available estimate of regional harvest for Central Africa, we find it very likely that, at a minimum, tens of millions of kilograms of wild meat (and wild animals) are wasted in Africa every year because of snaring.

## What can be done to address the negative impacts of snaring?

Snaring in Africa is therefore geographically widespread, locally intense, in many cases unsustainable, wasteful, and an animal welfare issue. What if anything, then, can be done to reduce the negative impacts of snaring? In the present article, we make three broad recommendations:

*First, in areas where hunting is legal, incentivize sustainable use at the local level through changes in governance.* Wire snaring is already illegal in many countries in Africa, but as we have shown, it remains pervasive. One of the most important challenges in managing snaring is that materials to make snares can be sourced from everyday objects (see above), making it virtually impossible to regulate access to snaring technology. Snares are also very difficult to detect, and even when they are detected it can be challenging to identify the owner, complicating enforcement. As a result, effectively managing snaring is unlikely to occur through top-down approaches alone and must include a focus on incentivizing sustainable practices among hunters themselves.

One way to incentivize sustainable use among hunters is through community governance (e.g., Esbach et al. 2024). Such systems can leverage collective policing and village-based social networks (e.g., hunters’ associations). They are also likely to be seen as legitimate among hunters and may be supported by strong preexisting norms that discourage rule breaking (Ostrom 1990). By tapping into these systems and norms, community governance has significant potential to reduce both overhunting and wastage in snaring. For example, communities often have strong social and cultural norms against unnecessary waste of food and other resources (Willcox and Nambu 2007, Jones 2020), so measures to reduce wastage and overhunting in snaring are likely to be well supported in many hunting societies.

There are also easy ways that hunters can reduce wastage. Previous studies reveal that probably the most important factor influencing discard rates is how often hunters check their snares. This is because animals caught in snares are more likely to rot or be scavenged with time. Discard rates can generally be reduced to 5% or less if hunters check their snares at least every 2 days (Vanwijnsberghe 1996, Kümpel 2006, Coad 2007). Importantly, checking snares at this rate would not necessarily increase the number of animals hunted (Froese et al. 2023). How often hunters check snares is itself influenced by several factors, including how far they set snares from villages (box 4), how many snares an individual hunter sets (Dounias 1993, Fialla Foffou 2011), and the time of the year: Snares set in larger numbers and at greater distances from villages are more likely to be checked less frequently, as are snares that are set during times of the year when farming or fishing are more labor intensive or lucrative (see box 2). In addition to how often hunters check snares, the type of material that hunters use to make snares probably affects wastage. Wire snares probably result in much more wastage than snares made of natural materials because they are more likely both to prevent animals from escaping and to leave animals with fatal injuries if they do escape. When abandoned, wire snares can also likely persist for years in the environment because of their durability, akin to ghost gear in fisheries (Macfadyen et al. 2009), adding to unnecessary mortality and wastage when animals become caught in them and die without being collected by hunters.

Box 4. **The relationship between distance of snares from villages and discard rates**

The distance at which hunters set snares from their villages, which we refer to as *distance*, can significantly affect discard rates. For example, some commercial hunters living in remote, forested regions of Central Africa set snares 15 to 20 kilometers or more from villages (because that is where valuable large game has yet to be overhunted and is therefore at higher densities; Amougou Mbatsogo 1998, Kümpel 2006, Yasuoka 2006, Abernethy et al. 2013, Koerner et al. 2017). To check these snares, hunters often camp in the forest for multiple days and visit snares regularly. However, some hunters do not deactivate their snares before returning to the village and can then leave their snares unchecked for weeks or months while staying in the village or traveling for social, financial (e.g., opportunities for paid work), or other reasons (e.g., illness). This can dramatically increase discard rates when they eventually return to their snares (Dethier 1995, 1996, Fotso and Ngnegueu 1997, Noss 1998, Muchaal and Ngandjui 1999, Kümpel 2006). Even when snares are within a day's walk from villages, distance can still affect discard rates simply because it takes more time and energy to check snares farther away (Amougou Mbatsogo 1998, Coad 2007).

**Figure 3.**
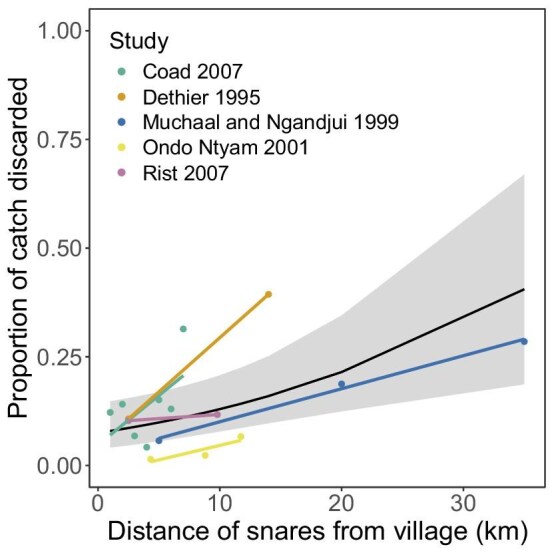
The distance of snares from a village is positively correlated with discard rates across five studies (GLMM: β = .061, σ = .020, p = .003). The black line and gray shaded ribbon are the modeled relationship with a 95% confidence interval in a generalized linear mixed model with study ID as a random effect. The color lines show individual trends within each study.

Several studies have measured discard rates at multiple distances from a village. To illustrate the relationship between distance and discard rates, we compiled data from these studies and modeled the relationship between distance and discards rates. Across studies, distance is strongly and positively correlated with discard rates (figure 3).

Through community governance, therefore, hunters could be incentivized to reduce wastage by using fewer snares, especially at certain times of the year, placing snares closer to villages, and using materials that break and decompose more readily. However, hunters are only likely to agree to these conditions if, while doing so, they can maintain catch of preferred species at desirable rates. Currently, many hunters set wire snares in large numbers far from villages (circumstances prone to result in wastage) because overhunting has depleted wildlife closer to villages (see box 4). Therefore, for hunters to regulate their use of snares in the ways suggested above, it will be essential to restore wildlife populations closer to villages and manage them at sufficiently high levels to incentivize self-regulation. This could be achieved through other actions supported by community governance including temporal or spatial hunting restrictions (Mockrin and Redford 2011, Griffiths et al. 2023, Esbach et al. 2024), forest management (Campera et al. 2019), and habitat restoration.

For community governance to be effective at reducing both wastage in snaring and overhunting, it will need to be supported by legal frameworks at higher levels of governance that recognize and secure the rights of communities to manage local resources.

This will almost certainly require the devolution of property rights to local communities, a cornerstone of common-pool resource governance (Ostrom 1990) and which has been key to successful community-based wildlife conservation in Namibia and Zimbabwe (Mavah et al. 2022). Given the current legislative insufficiencies in managing overhunting, as well as the importance of wild meat to livelihoods, diets, and culture (Rose 2000), some countries (e.g., the Democratic Republic of the Congo, Republic of the Congo, and Cameroon; https://www.swm-programme.info/legal-hub/) are already considering revising their hunting and trade legislation to balance the needs of people and wildlife. For these changes to be effective in managing snaring, they must focus not only on enforcement and regulation by national agencies but also on creating legal structures that enable effective community governance. At smaller levels, among communities themselves, community governance will likely need to include both face-to-face and community-wide participatory decision-making to increase accountability among community members and to prevent elite capture (Ostrom 2000, Ostrom 2007, Mavah et al. 2022).

*Second, continue or improve law enforcement and snare removal programs in protected areas where hunting is illegal.* In certain contexts, devolution may be considered inappropriate or politically unfeasible. For example, devolution may be unlikely to occur in national parks and other high-profile protected areas that contribute substantially to national GDPs and where hunting is illegal. In such cases, law enforcement and snare removal programs will help to manage snaring. For instance, there is evidence that sustained enforcement and removal programs can reduce both the numbers of snares in protected areas (African Parks 2019, 2020, Tilker et al. 2024) and injuries among wildlife (Mitani et al. 2024). These efforts will need to be supported by effective and fair judicial systems for snaring-related offenses, which in some countries may need reassessing and revising (Lindsey et al. 2013).

Enforcement and snare removal programs, however, require substantial financial resources and adequate staffing (African Parks 2019, 2020, Tilker et al. 2024), which can make them difficult to sustain. One way to obtain these resources in sufficient quantities is through delegated management models (a type of collaborative management model) in which nongovernmental organizations manage protected areas on behalf of national governments. These arrangements are particularly attractive to international donors and can clearly benefit wildlife populations (Lindsey et al. 2021, Denny et al. 2024).

It is also important to highlight that enforcement can have unintended impacts on local communities, including by altering conflict dynamics in conflict zones (Duffy et al. 2019, Denny et al. 2024). Therefore, if enforcement programs are to be viable in addressing snaring, they must be designed in ways that rigorously test for and address any negative social impacts. This will likely require substantial future research into how enforcement and protected area policies affect local communities within complex sociopolitical systems.

*Third, reduce demand for wild meat and livelihood dependence on hunting*. Any efforts to reduce the negative impacts of snaring must also address the underlying drivers of unsustainable hunting broadly. A full review of potential interventions and research needs to reduce unsustainable hunting is beyond the scope of this article; for a detailed review of these topics, we refer readers to Wicander and Coad (2018) and Coad and colleagues (2019). We briefly highlight, however, that combating unsustainable hunting will require developing locally appropriate and sustainable alternative food and income sources, as well as interventions that reduce demand for wild meat in places where it is being consumed at unsustainable levels (Coad et al. 2019). The latter will be especially important in urban environments, where wild meat consumption is often not a dietary necessity but where demand can be substantial (Ingram et al. 2021). To reduce demand, audience research can be used to characterize wild meat consumers and identify appropriate avenues for demand reduction messaging (Simo et al. 2024). Research can also help to identify whether providing dietary alternatives can reduce demand for wild meat in urban sites and, if so, whether increasing supply of these alternatives at urban locations can reduce hunting in rural areas (Wilkie et al. 2016). Where demand reduction efforts are deemed ethical and necessary for wildlife conservation, recent experimental evidence suggests that social marketing through information campaigns and community engagement (Chaves et al. 2018) and demand reduction videos (Cisse et al. 2025) may reduce wild meat consumption.

## Conclusions

Wildlife has immense intrinsic, instrumental, and relational value. But snaring has become a major threat to Africa's wildlife and its sustainable use, and it can be extremely wasteful from a conservation and food systems perspective. Limiting snaring will be difficult because, in many parts of Africa, the incentives to snare wildlife are high, and preventing access to technology to make snares is likely not possible. Nonetheless, there are opportunities to reduce the negative impacts of snaring. These include changes in governance that enable and incentivize the sustainable use of wildlife at the local level; snare removal programs and, where appropriate, improved enforcement; development projects in rural areas that reduce dependency on snaring for food and income; and demand reduction campaigns for wild meat, especially in urban areas, where wild meat is less likely to be consumed because of necessity. Not only will these measures reduce snaring and its impacts on wildlife and the environment, but they will also reduce wastage and support people who rely on wildlife for their food and livelihoods. Together, these measures provide paths toward achieving sustainability.

## Supplementary Material

biaf014_Supplemental_File

## Author Biography

Sean Denny (smdenny@bren.ucsb.edu) is affiliated with the Bren School of Environmental Science and Management, at the University of California, Santa Barbara, in Santa Barbara, California, in the United States. Lauren Coad is affiliated with the Center for International Forestry Research, in Bogor, Indonesia, and with the Department of Biology at the University of Oxford, in Oxford, England, in the United Kingdom. Sorrel Jones is affiliated with the RSPB Centre of Conservation Science, in Cambridge, England, in the United Kingdom. Daniel J. Ingram (d.j.ingram@kent.ac.uk) is affiliated with the Durrell Institute of Conservation and Ecology, in the School of Natural Sciences at the University of Kent, in Canterbury, England, in the United Kingdom.
