# Correction to: Snaring and wildlife wastage in Africa: drivers, scale, impacts, and paths to sustainability

**DOI:** 10.1093/biosci/biaf075

**Published:** 2025-06-09

Correction to: Sean Denny, Lauren Coad, Sorrel Jones, Daniel J Ingram, Snaring and wildlife wastage in Africa: drivers, scale, impacts, and paths to sustainability, *BioScience*, Volume 75, Issue 4, April 2025, Pages 284–297, https://doi.org/10.1093/biosci/biaf014

In the original publication, values from Fa et al. (2005)[i] were used to provide estimates for Central Africa of the mean body mass in kilograms (kg) of snared animals, and of the proportion of the total hunted catch that is caught using snares. These two numbers were used in the original publication to estimate the number of snares set (box 1), and the amount of wild meat wasted through snaring (box 3), annually in Central Africa.

Following discussions with the lead author of Fa et al. (2005), the authors determined that the estimates provided in Fa et al. (2005) were not suitable for this use. The authors have therefore used available open access datasets on hunting offtakes from the WILDMEAT database (www.wildmeat.org) to newly calculate the proportion of the overall catch which is caught using snares, and the average body mass of a snared animal. The authors have updated the estimates accordingly in the publication in boxes 1 and 3, and in the main text and abstract where these numbers are referenced.

The original estimate of the number of snares set annually in Central Africa was 27.8 million. This is now revised to an estimated range of 43.8 million to 234.5 million snares.

The original estimate of wasted wild meat per year in Central Africa was 46.9 million kg of mammalian wild meat. This is now revised to an estimated range of 10.9 million to 58.4 million kg including both mammalian and non-mammalian wild meat.

Further details of the datasets and calculation used to create these estimates have now been provided in an updated Supplementary material file.

The following reference has been also emended to read: “Coad L, Fa JE, Abernethy K, van Vliet N, Santamaria C, Wilkie D, El Bizri HR, Ingram DI, Cawthorn DM, Nasi R. 2019. Toward a Sustainable, Participatory and Inclusive Wild Meat Sector. Center for International Forestry Research (CIFOR).”

These changes do not affect the results, discussion and conclusions presented in the article. The published article has been updated.
