# Supplementary material for: Snaring and wildlife wastage in Africa: drivers, scale, impacts, and paths to sustainability
Source: Bioscience. 2025 Apr 9;75(4):284–97. doi: 10.1093/biosci/biaf014 (PMC12016803; doi:10.1093/biosci/biaf014)
Supplement: biaf014_Supplemental_File [file biaf014_supplemental_file.docx]

**Supplemental material S1**

**Snaring and wildlife wastage in Africa: drivers, scale, impacts, and paths to sustainability**

Sean Denny^1^*, Lauren Coad^2,3^, Sorrel Jones^4^, and Daniel J. Ingram^5^*

^1^ Bren School of Environmental Science and Management, University of California, Santa Barbara, Santa Barbara, CA, 93117, USA

^2^ Center for International Forestry Research (CIFOR), CIFOR Headquarters, Bogor 16115, Indonesia

^3^ Department of Biology, University of Oxford, 11a Mansfield Rd, Oxford OX1 3SZ, UK

^4^ RSPB Centre of Conservation Science, David Attenborough Building, Pembroke Street, Cambridge, CB2 3QZ, UK

^5^ Durrell Institute of Conservation and Ecology, School of Natural Sciences, University of Kent, Canterbury, CT2 7NR, UK

**Corresponding authors: Sean Denny (smdenny@bren.ucsb.edu) and Daniel J. Ingram (d.j.ingram@kent.ac.uk)*

Contents:

- Appendix 1: Further explanation of parameters and calculations in box 1.
- Table S1: Studies and data used to calculate average body masses of snared and shot animals, and the body mass ratio of snared to shot animals.
- Appendix 2: Further explanation of calculations in table 2.
- Appendix 3: Search methods for our literature review on discard and escape rates in snaring in Africa.
- Table S2: Studies included in our review of discard and escape rates in snaring in Africa.
- References cited.

**Appendix 1**. Further explanation of parameters and calculations in box 1.

In box 1, we use several parameters to estimate the number of snares set annually in Central Africa. Four key parameters are: (1) the proportion of the total number of animals caught that are snared, (2) the average body mass of animals caught with snares, (3) the ratio of the average body mass of animals caught with snares to the average body mass of animals caught with guns, and (4) the proportion of regional biomass (i.e., mass of wildlife) hunted annually in Central Africa that is attributable to snaring. Here, we justify and elaborate on how we calculated these parameters.

*Proportion of animals caught that are snared:*

To estimate this value, we drew on data from Ingram et al. (2025), who synthesized data from 83 studies from West and Central Africa to analyze regional hunting trends and patterns. Among other analyses, Ingram and colleagues (2025) modeled the proportion of animals hunted with guns versus all other hunting methods, and how this changed over time. Across studies conducted between 1991-2020 (the vast majority from Central Africa), they found that the proportion of animals hunted with guns increased from 25% to 76%. Importantly, this model was specific to village-based hunters (i.e., not forest hunter-gatherers), the temporal trend was only marginally significant, and their data did not include longitudinal studies (there is a dearth of such studies in the literature). Their model did, however, include the best available data on proportions of animals hunted with guns in Central Africa. For these reasons, and considering the large magnitude and spatial—indeed, regional—extent of the data underlying their analysis, we consider 25% to 76% a credible range for the proportion of animals hunted in Central Africa that are caught using guns.

To estimate the proportion of animals hunted that are caught with snares, we first took the complements of these percentages to generate a range for the proportion of animals hunted using any method other than gun hunting. This produced a range of 24% to 75% (100% - 76% and 100% - 25%, respectively). We then subtracted from this range an estimated proportion of animals that are hunted with methods other than snaring or gun hunting. Although the vast majority of animals in Central Africa are hunted with either snares or guns (Fa and Brown 2009, Coad et al. 2019), animals are also sometimes hunted with dogs, machetes, spears, and nets, among other methods. Several studies on village-based hunting indicate that the proportion of animals caught through these other methods is likely less than 10% or even less than 5% (Fa and García Yuste 2001, Wilcox and Nambu 2007, Froese et al. 2022). Therefore, we subtracted an additional 10% from the range above to conservatively account for animals caught via these other methods. This left a range of 14% to 65% for the proportion of animals snared (24% - 10% and 75% - 10%, respectively).

*Body masses of animals caught with snares, and the body mass ratio of animals caught with snares to animals caught with guns:*

To obtain regional estimates for these values we searched the WILDMEAT Use Database (<https://www.wildmeat.org/database/>) for studies that had open-access summary statistics or datasets on body masses of animals caught with snares or guns. We found four studies with available data (see table S1, below), which we used to calculate our body mass values and ratio. We also included Van Dijk 1999, which we knew had relevant data from our knowledge of the literature. Averaging values from these studies produced a mean body mass of 3.95 ± 2.58 kg for snared animals and a mean body mass of 5.32 ± 0.43 kg for animals hunted with guns. The ratio of these averages is 0.74 (3.95 kg ÷ 5.32 kg), which is within the range of study-specific ratios (0.48 to 0.85) (table S1).

*Proportion of total biomass that is attributable to snaring:*

To estimate the proportion of total biomass hunted annually in Central Africa that is attributable to snaring, we used the following equation:

$$\frac{proportion snared*mass ratio}{proportion snared*mass ratio+(\left( 1-proportion snared \right)*1)}$$

In this equation, *proportion snared* is the proportion of animals hunted annually in Central Africa that are caught with snares, and *mass ratio* is the ratio of the average body mass of snared animals to the average body mass of animals caught through means other than snaring (non-snared animals). By multiplying the proportion of animals that are snared by their average body mass relative to that of non-snared animals, the numerator captures the biomass contribution of snared animals relative to that of non-snared animals. The denominator represents the total estimated biomass of all hunted animals, combining the biomass of snared animals (weighted by their relative mass) and non-snared animals (weighted as 1, since the mass ratio is normalized to non-snared animals). Because there are few, if any, published body mass data for animals killed by hunting methods other than snaring or gun hunting, we use the mass ratio of snared to gun-hunted animals to be effectively equivalent to the mass ratio of snared to non-snared animals. We consider this to be reasonable, as the lack of studies that concern hunting with other methods also reflects the predominance of gun hunting and snaring observed across sites.

Using this equation, and the values generated above, we estimated a range for the proportion of total biomass attributable to snaring. Our lower estimate uses 14% for the proportion of hunted animals that are snared, and produces:

$$\frac{0.14*0.74}{0.14*0.74+\left( (1-0.14 \right)*1)}$$

= 0.108 or 10.8%.

Our upper estimate uses 65% for the proportion of hunted animals that are snared, and produces:

$$\frac{0.65*0.74}{0.65*0.74+\left( (1-0.65 \right)*1)}$$

= 0.579 or 57.9%.

**Table S1.** Studies and data used to calculate average body masses of snared and shot animals, and the body mass ratio of snared to shot animals.

| **Country** | **Study** | **Average body mass of snared animals (kg)** | **Average body mass of shot animals (kg)** | **Body mass ratio (kg) of snared to shot animals** |
| --- | --- | --- | --- | --- |
| Cameroon | Van Dijk 1999 | 2.8 | 5.8 | 0.48 |
| Central African Republic | Noss 1998^a^ | 8.21 | - | - |
| Equatorial Guinea | Vega et al. 2013^b^ | 1.64 | - | - |
| Equatorial Guinea | Kümpel 2006^c^ | 2.70 | 4.97 | 0.54 |
| Gabon | Coad 2005 | 4.4 | 5.2 | 0.85 |

^a^Noss (1998) reports the average body mass and number of individuals caught using snares, per species. We multiplied these values to generate total biomass of snared animals and then divided this by the total number of individuals caught.

^b^Vega et al. (2013) report average body masses of animals caught for two villages, per village. They do not report body masses by type of hunting method, but in one village all catch came from snaring. Therefore, we used the average body mass of animals caught from that village, Basilé Bubi.

^c^Kümpel (2006) reports body masses separately for animals shot, for animals caught using neck snares, and for animals caught using foot snares, as well as the number of animals caught using each method. To generate an average body mass for snaring broadly, for each snaring method we multiplied the number of animals caught by the average mass of animals caught. We then summed these values to create a total biomass of animals caught through snaring and divided it by the total number of animals snared.

**Appendix 2**. Further explanation of calculations in table 2.

For each dataset of protected areas, ‘snares removed per protected area per year (average)’ was calculated by dividing ‘total snares removed across all protected areas’ by ‘total protected area-years’, and rounding to the nearest whole number. ‘Snares removed per km^2^ per year (average)’ was calculated by multiplying ‘snares removed per protected area per year (average)’ by ‘number of protected areas’ (i.e., by 11), dividing by ‘total combined area of protected areas (km^2^)’, and rounding to the nearest thousandth decimal place. For example, for our dataset for Africa, we multiplied 4908 snares removed per protected area per year by 11 protected areas to generate 53,988 snares removed across all 11 protected areas per year. We then divided this number by the total size of the 11 protected areas—38,504 km^2^—to obtain 1.402 snares removed per km^2^ per year on average.

**Appendix 3**. Search methods for our literature review on discard and escape rates in snaring in Africa.

We identified discard and escape rates by first searching the scientific literature in Ingram (2018) and the WILDMEAT Library ([www.wildmeat.org/publications](http://www.wildmeat.org/publications/)), which were originally found using systematic searches. We then conducted searches using Google Scholar to identify recently published papers. We used the search terms *wild meat, wildmeat, bushmeat, hunting, snar*, trap**, *discard*, wast**, *rott**, and *escap**. Finally, we conducted snowball sampling through the reference lists of identified papers to find other published or gray literature. When reviewing literature, we defined discard rates as the number of animals discarded out of the total number of animals killed by snares, and escape rates as the number of animals that escaped from snares out of the total number of animals caught in snares (i.e., the sum of those that were killed and those that escaped).

**Table S2.** Studies included in our review of discard and escape rates in snaring in Africa (illustrated in figure 2 of the main text). Studies are arranged in alphabetical order, first by country, then by author within country, and finally by year.

| **Country where the study took place** | **Study** |
| --- | --- |
| Cameroon | Abugiche 2008 |
| Cameroon | Dethier 1995 |
| Cameroon | Dounias 1993 |
| Cameroon | Fialla Foffou 2011 |
| Cameroon | Fotso and Ngnegueu 1997 |
| Cameroon | Ngueguim 2001 |
| Cameroon | Nguetsop 2001 |
| Cameroon | Ondo Ntyam 2001 |
| Cameroon | Willcox and Nambu 2007 |
| Cameroon | Yasuoka 2006 |
| Central African Republic | Dethier 1996 |
| Central African Republic | Noss 1998 |
| Democratic Republic of the Congo | Brown 2007 |
| Equatorial Guinea | Allebone-Webb 2009 |
| Equatorial Guinea | Fa and García Yuste 2001 |
| Equatorial Guinea | Kümpel 2006 |
| Equatorial Guinea | Rist 2007 |
| Gabon | Coad 2007 |
| Gabon | Coad 2013 (unpublished data) |
| Liberia | Greengrass 2011 |
| Liberia | Jones et al. 2020 |
| Madagascar | Borgerson 2016 |
| Republic of the Congo | Vanwijnsberghe 1996 |
| Tanzania | Nielsen 2006 |

**References cited**

Abugiche SA. 2008. Impact of Hunting and Bushmeat Trade on Biodiversity Loss in Cameroon: A Case Study of the Banyang-Mbo Wildlife Sanctuary. PhD dissertation. Brandenburg University of Technology in Cottbus.

Allebone-Webb SM. 2009. Evaluating dependence on wildlife products in rural Equatorial Guinea. PhD dissertation. Imperial College London; Zoological Society of London.

Belecky M, Gray TNE. 2020. Silence of the Snares: Southeast Asia’s Snaring Crisis. WWF International.

Borgerson C. 2016. Optimizing conservation policy: the importance of seasonal variation in hunting and meat consumption on the Masoala Peninsula of Madagascar. Oryx 50: 405–418.

Brown E. 2007. Managing Hunting and Transportation of Wildlife In and Around the Okapi Faunal Reserve, Democratic Republic of Congo. WCS Okapi Faunal Reserve, DRC.

Coad L. 2005. Individual hunting data from two villages in Gabon collected between 2003 and 2005. Accessed from the WILDMEAT database on April 20 2025. https://explorer.wildmeat.org/study/13

Coad L. 2007. Bushmeat hunting in Gabon: Socio-economics and hunter behaviour. PhD dissertation. Emmanual College, University of Cambridge; Imperial College London.

Coad L, Fa JE, Abernethy K, van Vliet N, Santamaria C, Wilkie D, El Bizri HR, Ingram DI, Cawthorn DM, Nasi R. 2019. Toward a Sustainable, Participatory and Inclusive Wild Meat Sector. Center for International Forestry Research (CIFOR).

Dethier M. 1995. Etude chasse. Ministère de l’Environnement, Cameroun.

Dethier M. 1996. Etude chasse villageoise Forêt de N’gotto. Ministère des Eaux et Forêts, République Centrafricaine.

Dounias E. 1993. Dynamique et gestion differentielles du systeme de production a dominante agricole des Mvae du Sud-Cameroun forestier. PhD dissertation. University of Montpelier II, Montpelier, France.

Fa JE, Brown D. 2009. Impacts of hunting on mammals in African tropical moist forests: A review and synthesis. Mammal Review 39: 231–264.

Fa JE, García Yuste JE. 2001. Commercial bushmeat hunting in the Monte Mitra forests, Equatorial Guinea: extent and impact. Animal Biodiversity and Conservation 24: 31–52.

Fialla Foffou JC. 2011. Évaluation de la pression au braconnage sur la faune dans le parc national de Campo-Ma’an et sa zone périphérique. Mémoire de Master. Université de Yaoundé 1, Yaoundé, Cameroun.

Fotso RC, Ngnegueu PR. 1997. Commercial hunting and its consequences on the dynamic of duiker populations. Ministère de l’Environnement, Cameroun.

Froese GZL, et al. 2022. Coupling paraecology and hunter GPS self-follows to quantify village bushmeat hunting dynamics across the landscape scale. African Journal of Ecology 60: 229-249.

Greengrass E. 2011. Exploring the dynamics of bushmeat hunting and trade in Sapo National Park.

Ingram DJ, et al. 2025. Regional patterns of wild animal hunting in African tropical forests. Nature Sustainability (2025): s41893-024-01494-5. https://doi.org/10.1038/s41893-024-01494-5.

Jones SCZ, Papworth SK, St. John FAV, Vickery JA, Keane AM. 2020. Consequences of survey method for estimating hunters’ harvest rates. Conservation Science and Practice 2.

Kümpel NF. 2006. Incentives for sustainable hunting of bushmeat in Río Muni, Equatorial Guinea. Ph.D. dissertation. Imperial College London; Zoological Society of London.

Ngueguim JR. 2001. Etude de la chasse villageoise dans l’unite technique operationnelle Campo-Ma’an: cas du secteur Bifa.

Nguetsop JD. 2001. Etude de la chasse villageoise dans la peripherie sud ouest de l’unite technique operationnelle de Campo-Ma’an: cas du secteur Campo.

Nielsen MR. 2006. Importance, cause and effect of bushmeat hunting in the Udzungwa Mountains, Tanzania: Implications for community based wildlife management. Biological Conservation 128: 509–516.

Noss AJ. 1998. The Impacts of Cable Snare Hunting on Wildlife Populations in the Forests of the Central African Republic. Conservation Biology 12: 9.

Ondo Ntyam SC. 2001. Etude de la chasse villageoise dans la peripherie sud ouest de l’unite technique operationnelle de Campo-Ma’an: cas des villages.

Rist J. 2007. Bushmeat Catch per Unit Effort in space and time: a monitoring tool for bushmeat hunting. PhD dissertation. Imperial College London.

van Dijk JFW. 1999. Non-Timber Forest Products in the Bipindi-Akom II Region, Cameroon: A socio-economic and ecological assessment. The Tropenbos-Cameroon programme.

Vanwijnsberghe S. 1996. Etude sur la chasse villageoise aux environs au Parc National d’Odzala. Ministère de l’Agriculture, de l’Elevage, des Eaux et Forêts et des Ressources Halieutiques.

Vega MG, Carpinetti B, Duarte J, Fa JE. 2013. Contrasts in livelihoods and protein intake between commercial and subsistence bushmeat hunters in two villages on Bioko Island, Equatorial Guinea. Conservation Biology 27: 576-587.

Willcox AS, Nambu DM. 2007. Wildlife hunting practices and bushmeat dynamics of the Banyangi and Mbo people of Southwestern Cameroon. Biological Conservation 134: 251–261.

Yasuoka H. 2006. The sustainability of duiker (Cephalophus spp.) hunting for the Baka hunter-gatherers in southeastern Cameroon. African Study Monographs 33: 95–120.
